# Characterization bacterial metabolites and peripheral immune cell populations in stable and progressive Alzheimer’s disease

**DOI:** 10.1002/alz.092564

**Published:** 2025-01-03

**Authors:** Ethan B Loew, Matthew Tracy, Cynthia Jo, Beth McCormick, John P Haran

**Affiliations:** ^1^ University of Massachusetts Chan Medical School, Worcester, MA USA; ^2^ University of Massachusetts Chan Medical School Chan Medical School, Worcester, MA USA

## Abstract

**Background:**

Alzheimer’s disease (AD) is the most common type of dementia which results in debilitating memory loss as the disease advances. However, among older adults with AD, some may experience rapid cognitive decline while others may maintain a stable cognitive status for years. In addition to the amyloid plaques, tau tangles, and neuronal inflammation characteristic of AD, there is strong evidence of dysregulation in the peripheral immune system, including decreased naïve T cells and increased memory T cells among older adults with AD. It is currently unknown what underlies dysfunction in the peripheral immune system or whether changes in peripheral immune cells are associated with cognitive decline.

**Method:**

We have performed unbiased metabolomics and characterized stool metabolites present in 35 AD versus 35 propensity matched healthy controls. In our ongoing work, we are longitudinally characterizing resting peripheral immune cell populations by flow cytometry and gut microbiome composition by metagenomic sequencing.

**Result:**

We have identified an increase in the metabolites methionine sulfone (1.46 fold, p<0.05), homocysteine (1.67 fold, p<0.05), and cysteine (1.33 fold, p<0.05) in the stool of older adults with AD compared to controls. Among the population of AD patients experiencing cognitive decline, determined by increasing ADAS‐Cog score >6 points over one year (n = 7 declining vs n = 8 stable cognition), we have identified increases in the bacterial genes responsible for methionine production at the point of cognitive decline compared to previous timepoints and between patients with decline versus stable cognition. In accordance with the role of methionine in promoting immune cell proliferation and differentiation, we have compared the composition of peripheral immune cells among adults with declining versus stable cognition and identified a decrease in CD4^+^/CD62L^+^ naïve T cells (percent of CD4^+^ lymphocytes, stable 0.3055 vs declining 0.0955, p = 0.0042) and increased effector memory CD4^+^ T cells (percent of CD4^+^ lymphocytes, stable = 0.2375 vs declining = 0.4164, p = 0.0225).

**Conclusion:**

This longitudinal clinical study identifies changes in stool metabolites and resting peripheral T cell populations in AD patients and among AD patients with cognitive decline. We propose that gut bacterial produced methionine acts to promote peripheral immune differentiation and dysfunction, leading to cognitive decline in AD.

